# Development and validation of a machine learning-based prognostic risk stratification model for acute ischemic stroke

**DOI:** 10.1038/s41598-023-40411-2

**Published:** 2023-08-23

**Authors:** Kai Wang, Tao Hong, Wencai Liu, Chan Xu, Chengliang Yin, Haiyan Liu, Xiu’e Wei, Shi-Nan Wu, Wenle Li, Liangqun Rong

**Affiliations:** 1 Department of Neurology, The Second Affiliated Hospital of Xuzhou Medical University, Xuzhou, Jiangsu China; 2 Key Laboatory of Neurological Diseases, The Second Affiliated Hospital of Xuzhou Medical University, Xuzhou, Jiangsu China; 3 Pediatric Surgery Ward, Fuwai Hospital Chinese Academy of Medical Sciences, Shenzhen, China; 4 Department of Cardiovascular Surgery, General Hospital of Northern Theater Command, Shenyang, 110000 China; 5 Postgraduate College, Dalian Medical University, Dalian, 116000 China; 6 Department of Orthopaedics, Shanghai Jiao Tong University Affiliated Sixth People’s Hospital, 600 Yishan Road, Shanghai, 200233 China; 7 The State Key Laboratory of Molecular Vaccinology and Molecular Diagnostics & Center for Molecular Imaging and Translational Medicine, School of Public Health, Xiamen University, Xiamen, China; 8 Faculty of Medicine, Macau University of Science and Technology, Macau, China; 9 School of Medicine, Eye Institute of Xiamen University, Xiamen University, Xiamen, Fujian China

**Keywords:** Neurology, Neurological disorders

## Abstract

Acute ischemic stroke (AIS) is a most prevalent cause of serious long-term disability worldwide. Accurate prediction of stroke prognosis is highly valuable for effective intervention and treatment. As such, the present retrospective study aims to provide a reliable machine learning-based model for prognosis prediction in AIS patients. Data from AIS patients were collected retrospectively from the Second Affiliated Hospital of Xuzhou Medical University between August 2017 and July 2019. Independent prognostic factors were identified by univariate and multivariate logistic analysis and used to develop machine learning (ML) models. The ML model performance was assessed by area under the receiver operating characteristic curve (AUC) and radar plot. Shapley Additive explanations (SHAP) values were used to interpret the importance of all features included in the predictive model. A total of 677 AIS patients were included in the present study. Poor prognosis was observed in 209 patients (30.9%). Six variables, including neuron specific enolase (NSE), homocysteine (HCY), S-100β, dysphagia, C-reactive protein (CRP), and anticoagulation were included to establish ML models. Six different ML algorithms were tested, and Random Forest model was selected as the final predictive model with the greatest AUC of 0.908. Moreover, according to SHAP results, NSE impacted the predictive model the most, followed by HCY, S-100β, dysphagia, CRP and anticoagulation. Based on the RF model, an online tool was constructed to predict the prognosis of AIS patients and assist clinicians in optimizing patient treatment. The present study revealed that NSE, HCY, CRP, S-100β, anticoagulation, and dysphagia were important factors for poor prognosis in AIS patients. ML algorithms were used to develop predictive models for predicting the prognosis of AIS patients, with the RF model presenting the optimal performance.

## Introduction

Acute ischemic stroke (AIS), is the fifth leading cause of death in the United States^1^, and the leading cause of death in China^2^. AIS imposes a very heavy burden on high-income countries with the rapid development of social economy. Furthermore, the corresponding burden is increasing rapidly in low-income and middle-income countries^3^. The identification of risk factors associated with poor prognosis in AIS patients could assist clinicians to provide close surveillance and timely intervention for high-risk stroke patients with poor prognosis and may guide the implementation of strategies in organized stroke care provision, thereby optimizing clinical outcomes. Machine learning (ML) algorithms can efficiently identify features highly correlated to outcomes from a large number of features, outperforming traditional statistical methods^4,5^. Consequently, ML can be used to improve the prediction accuracy of prognosis.

ML algorithms using big data have improved and optimized the predictive performance of the prognosis prediction, as reported by prior literature^6–8^. Therefore, the goal in this retrospective study was to identify factors associated with prognosis for patients with stroke and develop an ML-based predictive model. Moreover, the proposed model was integrated in an online tool to provide clinicians and patients with visual and practical predictive assessment.

## Materials and methods

### Data source and collection

Retrospective data were obtained from electronic health record of the Second Affiliated Hospital of Xuzhou Medical University. Patients diagnosed with AIS from August 2017 to July 2019 were enrolled in this study cohort. Inclusion criteria were: The diagnosis of AIS met the requirements of the World Health Organization with symptom onset less than 24 h^9^. Exclusion criteria: (1) Incomplete clinical data. (2) Patients with severe abnormal organ function. (3) Inadequate auxiliary examinations. (4) Follow-up less than one year. The diagnosis process of AIS was completed and confirmed independently by two physicians. In situations where there was diagnostic disagreement, the final diagnosis was reviewed with a senior physician to reach a consensus. This study was approved by the Ethics Committee of the Second Affiliated Hospital of Xuzhou Medical University, and all studies were conducted in accordance with relevant guidelines/regulations and the Declaration of Helsinki, and informed consent was obtained from all participants.

### Data processing and variable selection

**Figure 1:**
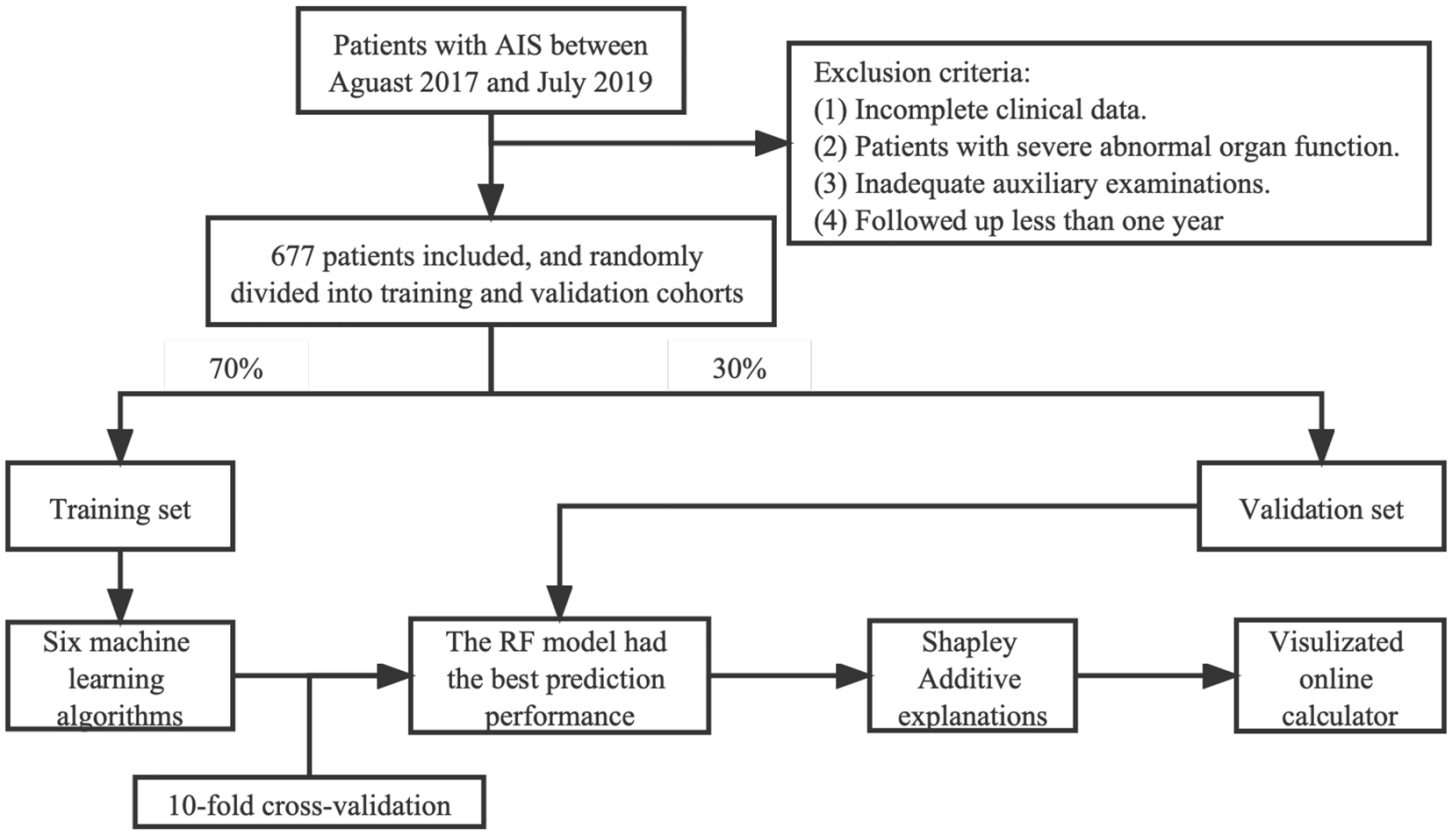
The flowchart of the patient selection.

In the data cleaning process, the information of subjects with missing value was deleted, and normality tests were performed for the continuous numerical variables to assess the presence of outliers. Subsequently, the information of subjects with outliers was deleted. Ultimately, the current study included a total of 677 subjects in the database. The data were collected by trained and qualified members of the research group using a uniformly designed questionnaire, which included living area, occupation, education level, family economic status, eating habits (alcohol intake, drinking sugary drinks, smoking, high-fat diet, etc.), lifestyle and habits, disease medication, menstrual history (female) and psychosocial factors. First, the purpose and significance of the survey were explained to the patients. On the basis of patients fully understanding, informed consent forms were signed, and the patients were asked to read the guidance on the questionnaire carefully and fill it in. Patients with reading difficulties, such as illiteracy or poor eyesight, were aided by members of the research group and the contents of the questionnaire were read to them to help them fill it in. The type and severity of stroke were evaluated by clinical symptoms, head CT, MR, and angiography results. Blood lipid, blood glucose, homocysteine, and other data were obtained by laboratory tests. Blood pressure, weight, height, time of onset, and baseline National Institutes of Health Stroke Scale (NIHSS) score were also recorded. Drug use, stroke recurrence and clinical prognosis were recorded during follow-up. Dysphagia was assess by water swallowing test according to the criteria^10^. Clinical prognosis was assessed according to the modified Rankin Scale (mRS) according to the results of 1-year follow-up. The mRS ≥ 3 was classified as poor prognosis and mRS < 3 was classified as good prognosis. All detection indexes were completed by the Second Hospital of Xuzhou Medical University. The flowchart of data collection is shown in Fig. 1.

Binary categorical features were encoded with 0 and 1; for example, the gender of patients was encoded as 0 or 1 (0 = male, 1 = female). A total of 30 demographic and clinical variables were included as baseline variables for analysis. All parameter data were retrieved from inpatient electronic medical records. Univariate logistic analysis was performed to identify impactful clinical variables, and then statistically significant variables were included in multivariate logistic analysis.

### Model development and performance evaluation

This dataset was randomly split into a training cohort (70%) and a validation cohort (30%). The training cohort was used to construct and perform cross-validated ML models to avoid overfitting, and the validation database was used to validate the predictive power of models.

Based on the results of multivariate logistic analysis, the identified statistically significant variables were used to construct ML models. Six ML algorithms were employed to develop predictive models based on the training cohort, the performance of all candidate models was assessed using tenfold cross-validation within the training cohort, the final model was identified according to the highest mean AUC and the performance was further validated on the validation cohort. During tenfold cross-validation, the training cohort was divided into ten sets, nine of them were used for model testing and one for model assessment. Meanwhile, radar plots were drawn and the accuracy, sensitivity and specificity were calculated to evaluate performance. Due to the “black-box” nature of ML algorithms, the Shapley Additive explanations (SHAP) metric was used to interpret the models and assist doctors understand the findings of the models. The contribution of each variable to the model prediction was evaluated by SHAP values^11,12^.

### Statistical analysis

Continuous and categorical variables were expressed as mean ± SD and frequency, respectively. P-Values < 0.05 were considered statistically significant with 95% confidence intervals (CIs) applied for all analyses. Statistical analyses of the demographic and clinical characteristics of all included patients were performed in R (version 4.0.5, HTTPS: //www.r-project.org/). Python (version 3.8) was used to develop ML predictive models and a web risk calculator.

### Ethics approval and consent to participate

This study was approved by the Ethics Committee of the Second Affiliated Hospital of Xuzhou Medical University (ethics number: [2020] 081603), and all studies were conducted in accordance with relevant guidelines/regulations, informed consent was obtained from all participants, and all studies were conducted in accordance with the Declaration of Helsinki.

## Results

### Characteristics of study population

A total of 713 AIS patients were included in this study, and 36 patients with missing value were deleted. Finally, 677 patients diagnosed as AIS were enrolled in the present study, and 209 patients had poor prognosis (30.9%). The differences between AIS patients with good and poor prognosis are described in Table 1. No differences in stroke occurrence between age and gender groups were observed, and there were no significant differences in the location of stroke-associated arterial blood vessels.

**Table 1:** Baseline characteristics of AIS patients between good and poor prognosis.

Characteristics		Overall	Good	Poor	P-value
N		677	468	209	
Age, n (%)	< 60	383 (56.6)	265 (56.6)	118 (56.5)	0.965
	≥ 60	294 (43.4)	203 (43.4)	91 (43.5)	
Gender, n (%)	Female	279 (41.2)	183 (39.1)	96 (45.9)	0.113
	Male	398 (58.8)	285 (60.9)	113 (54.1)	
SBP, median [Q1, Q3]		143.0 [132.0,156.0]	139.0 [130.0,152.2]	149.0 [138.0,166.0]	< 0.001
DBP, median [Q1, Q3]		87.0 [74.0,97.0]	84.0 [71.8,94.2]	90.0 [83.0,99.0]	< 0.001
SD, n (%)	Anterior	270 (39.9)	174 (37.2)	96 (45.9)	0.054
	Posterior	252 (37.2)	177 (37.8)	75 (35.9)	
	Anterior/Posterior	155 (22.9)	117 (25.0)	38 (18.2)	
SOH, n (%)	Left	283 (41.8)	195 (41.7)	88 (42.1)	0.765
	Right	270 (39.9)	184 (39.3)	86 (41.1)	
	Bilateral	124 (18.3)	89 (19.0)	35 (16.7)	
SOS, n (%)	Cortex	155 (22.9)	111 (23.7)	44 (21.1)	0.308
	Cortex-subcortex	155 (22.9)	107 (22.9)	48 (23.0)	
	Subcortex	186 (27.5)	121 (25.9)	65 (31.1)	
	Brainstem	104 (15.4)	79 (16.9)	25 (12.0)	
	Cerebellum	77 (11.4)	50 (10.7)	27 (12.9)	
NOS, n (%)	Single lesion	470 (69.4)	319 (68.2)	151 (72.2)	0.329
	Multiple lesions	207 (30.6)	149 (31.8)	58 (27.8)	
Cholesterol, median [Q1, Q3]		5.3 [4.4,6.2]	5.2 [4.4,6.1]	5.7 [4.5,6.2]	0.005
Triglyceride, median [Q1, Q3]		2.2 [1.9,2.4]	2.2 [1.9,2.4]	2.2 [1.9,2.4]	0.28
LDL, median [Q1,Q3]		4.8 [4.3,4.9]	4.7 [4.4,4.9]	4.8 [4.3,4.9]	0.796
FBG, median [Q1, Q3]		5.3 [4.6,5.8]	5.2 [4.6,5.8]	5.3 [4.7,6.0]	0.142
HBALC, median [Q1, Q3]		5.6 [5.3,5.9]	5.6 [5.3,5.9]	5.7 [5.4,6.0]	0.055
HCY, median [Q1, Q3]		15.8 [12.7,19.4]	15.0 [12.2,18.5]	18.2 [13.9,20.8]	< 0.001
UA, median [Q1, Q3]		349.8 [309.8,408.1]	346.9 [310.7,403.3]	357.8 [302.9,423.9]	0.261
MB, median [Q1, Q3]		97.7 [75.1,147.8]	96.8 [76.5,137.2]	98.6 [69.8,181.1]	0.106
CRP, median [Q1, Q3]		12.6 [7.7,17.6]	10.8 [7.2,15.3]	17.1 [9.6,21.4]	< 0.001
FIB, median [Q1, Q3]		4.3 [4.0,4.8]	4.4 [4.0,4.8]	4.3 [3.8,4.7]	0.055
Ddimer, median [Q1, Q3]		174.0 [133.0,221.0]	175.0 [131.0,227.2]	170.0 [137.0,212.0]	0.284
BNP, median [Q1, Q3]		93.0 [73.0,162.0]	93.0 [74.0,163.0]	93.0 [71.0,157.0]	0.707
NSE, median [Q1, Q3]		16.2 [12.7,18.6]	14.5 [12.3,17.2]	18.7 [17.6,20.1]	< 0.001
S100β, median [Q1, Q3]		275.0 [224.0,290.0]	268.0 [217.8,284.0]	292.0 [259.0,312.0]	< 0.001
Thrombolysis, n (%)	No	473 (69.9)	346 (73.9)	127 (60.8)	0.001
	Yes	204 (30.1)	122 (26.1)	82 (39.2)	
Thrombectomy, n (%)	No	644 (95.1)	456 (97.4)	188 (90.0)	< 0.001
	Yes	33 (4.9)	12 (2.6)	21 (10.0)	
Antiplatelet, n (%)	No	122 (18.0)	101 (21.6)	21 (10.0)	< 0.001
	Yes	555 (82.0)	367 (78.4)	188 (90.0)	
Anticoagulation, n (%)	No	576 (85.1)	423 (90.4)	153 (73.2)	< 0.001
	Yes	101 (14.9)	45 (9.6)	56 (26.8)	
Statin, n (%)	No	103 (15.2)	84 (17.9)	19 (9.1)	0.004
	Yes	574 (84.8)	384 (82.1)	190 (90.9)	
PPI, n (%)	No	535 (79.0)	401 (85.7)	134 (64.1)	< 0.001
	Yes	142 (21.0)	67 (14.3)	75 (35.9)	
Dysphagia, n (%)	No	525 (77.5)	410 (87.6)	115 (55.0)	< 0.001
	Yes	152 (22.5)	58 (12.4)	94 (45.0)	
SAP, n (%)	No	512 (75.6)	393 (84.0)	119 (56.9)	< 0.001
	Yes	165 (24.4)	75 (16.0)	90 (43.1)	

*SBP* systolic blood pressure, *DBP* diastolic blood pressure, *SD* stroke distribution, *SOH* side of hemisphere, *SOS* site of stroke lesions, *NOS* number of stroke lesions, *LDL* low-density lipoprotein, *FBG* fasting blood glucose, *HCY* homocysteine, *UA* uric acid, *MB* myoglobin, *CRP* C-reactive protein, *FIB* fibrinogen, *BNP* brain natriuretic peptide, NSE neuron-specific enolase, *PPI* proton pump inhibitor therapy, *SAP* stroke-associated pneumonia.

### Correlation of variables with clinical outcome

A total of 474 patients were assigned to the training cohort (70% of the total population). In the training cohort, univariable regression was used to identify fifteen significant risk factors, including Systolic Blood Pressure (SBP), Diastolic Blood Pressure (DBP), Homocysteine (HCY), Myoglobin (MB), C-reactive protein (CRP), Neuron-Specific Enolase (NSE), S100β, treatment history (thrombolysis, thrombectomy, antiplatelet, anticoagulation, statin, PPI), and complicates (dysphagia and stroke-associated pneumonia) (Table 2). Subsequently, the previously identified variables were used to determine independent risk factors using multivariate regression. The results of the multivariable analysis in the training cohort are presented in Table 2. Independent risk factors associated with prognosis included NSE, HCY, CRP, S-100β, dysphagia, and anticoagulation.

**Table 2:** Univariate and multivariate logistic regression analysis of risk factors for poor prognosis in AIS patients.

Characteristics	Univariate logistic analysis			Multivariate logistic analysis		
	OR	95%CI	P-Value	OR	95%CI	P-Value
Age						
< 60		Ref	Ref			
≥ 60	1.01	(0.72–1.40)	0.967			
Gender						
Female		Ref	Ref			
Male	0.76	(0.54–1.05)	0.097			
SBP	1.03	(1.02–1.04)	< 0.001	1.01	(0.99–1.03)	0.302
DBP	1.03	(1.02–1.05)	< 0.001	1	(0.97–1.03)	0.979
SD						
Anterior		Ref	Ref		Ref	Ref
Posterior	0.77	(0.53–1.11)	0.16	0.67	(0.4–1.11)	0.12
Anterior/Posterior	0.59	(0.38–0.91)	0.018	0.64	(0.35–1.15)	0.138
SOH						
Left		Ref	Ref			
Right	1.04	(0.72–1.48)	0.849			
Bilateral	0.87	(0.54–1.38)	0.567			
SOS						
Cortex		Ref	Ref			
Cortex-subcortex	1.13	(0.69–1.85)	0.622			
Subcortex	1.35	(0.85–2.16)	0.199			
Brainstem	0.8	(0.45–1.41)	0.444			
Cerebellum	1.36	(0.75–2.44)	0.304			
NOS						
Single lesion		Ref	Ref			
Multiple lesions	0.82	(0.57–1.18)	0.288			
Cholesterol	1.21	(1.06–1.38)	0.004	1.12	(0.94–1.34)	0.203
Triglyceride	1.27	(0.81–2.00)	0.297			
LDL	1.09	(0.87–1.37)	0.441			
FBG	1.14	(0.97–1.36)	0.121			
HBALC	1.51	(1.01–2.26)	0.045	1.25	(0.72–2.17)	0.421
HCY	1.17	(1.12–1.22)	< 0.001	1.24	(1.16–1.33)	< 0.001
UA	1	(1.00–1.00)	0.209			
MB	1	(1.00–1.01)	< 0.001	1	(1–1)	0.421
CRP	1.12	(1.09–1.15)	< 0.001	1.05	(1.02–1.09)	0.004
FIB	0.77	(0.59–1.00)	0.048	0.85	(0.59–1.23)	0.394
Ddimer	1	(1.00–1.00)	0.45			
BNP	1	(1.00–1.00)	0.94			
NSE	1.45	(1.36–1.55)	< 0.001	1.38	(1.27–1.51)	< 0.001
S100β	1.02	(1.01–1.02)	< 0.001	1.01	(1–1.01)	0.005
Thrombolysis						
No		Ref	Ref		Ref	Ref
Yes	1.83	(1.29–2.59)	0.001	0.78	(0.46–1.32)	0.358
Thrombectomy						
No		Ref	Ref		Ref	Ref
Yes	4.21	(2.05–9.05)	< 0.001	0.5	(0.16–1.55)	0.226
Antiplatelet						
No		Ref	Ref		Ref	Ref
Yes	2.45	(1.51–4.15)	< 0.001	1.04	(0.42–2.6)	0.932
Anticoagulation						
No		Ref	Ref		Ref	Ref
Yes	3.43	(2.23–5.32)	< 0.001	1.91	(1.03–3.56)	0.041
Statin						
No		Ref	Ref		Ref	Ref
Yes	2.17	(1.31–3.79)	0.002	1.42	(0.55–3.66)	0.464
PPI						
No		Ref	Ref		Ref	Ref
Yes	3.34	(2.28–4.92)	< 0.001	1.47	(0.8–2.68)	0.21
Dysphagia						
No		Ref	Ref		Ref	Ref
Yes	5.75	(3.92–8.52)	< 0.001	3.77	(2.13–6.7)	< 0.001
SAP						
No		Ref	Ref		Ref	Ref
Yes	3.95	(2.74–5.73)	< 0.001	1.54	(0.85–2.78)	0.157

*SBP* systolic blood pressure, *DBP* diastolic blood pressure, *SD* stroke distribution, *SOH* side of hemisphere, *SOS* site of stroke lesions, *NOS* number of stroke lesions, *LDL* low-density lipoprotein, *FBG* fasting blood glucose, *HCY* homocysteine, *UA* uric acid, *MB* myoglobin, *CRP* C-reactive protein, *FIB* fibrinogen, *BNP* brain natriuretic peptide, *NSE* neuron-specific enolase, *PPI* proton pump inhibitor therapy, *SAP* stroke-associated pneumonia.

### Development and validation of predictive models

**Figure 2:**
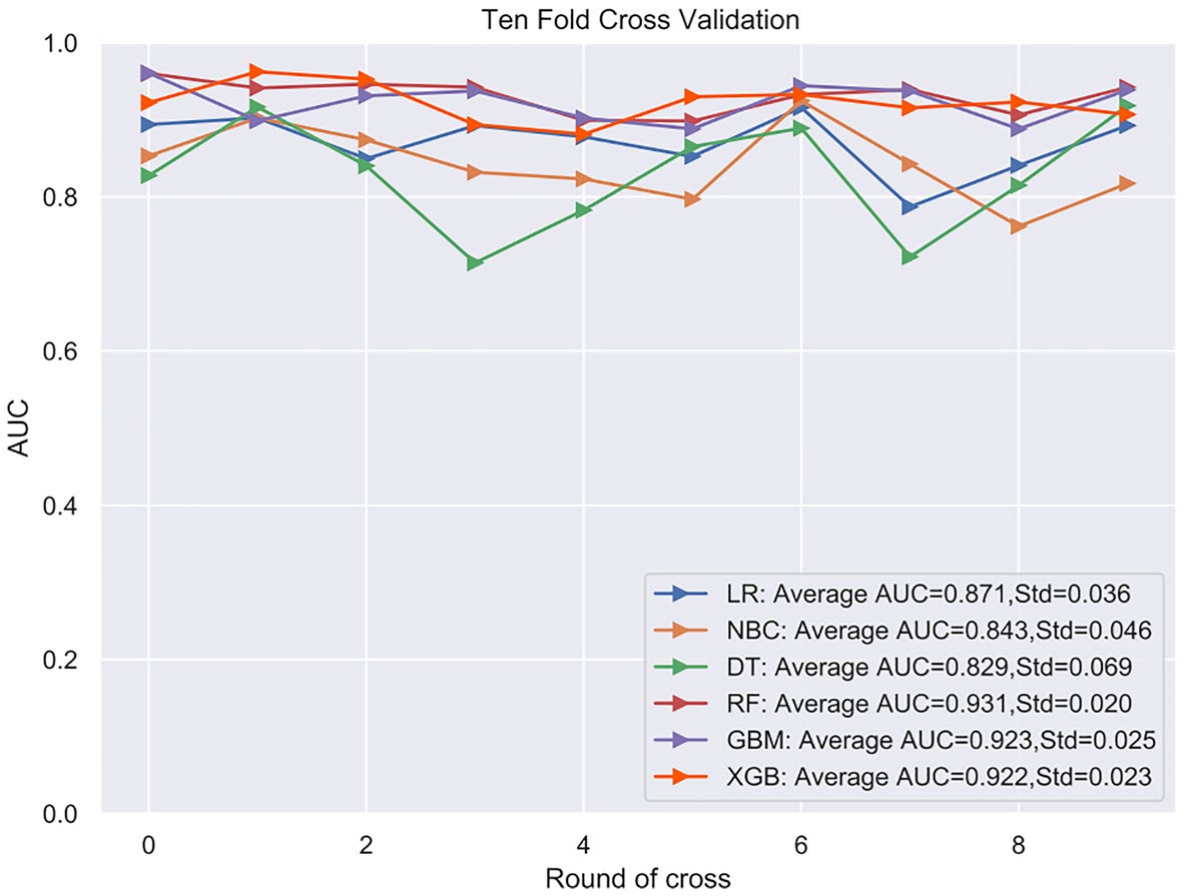
The results of tenfold cross-validation in the training cohort.

**Figure 3:**
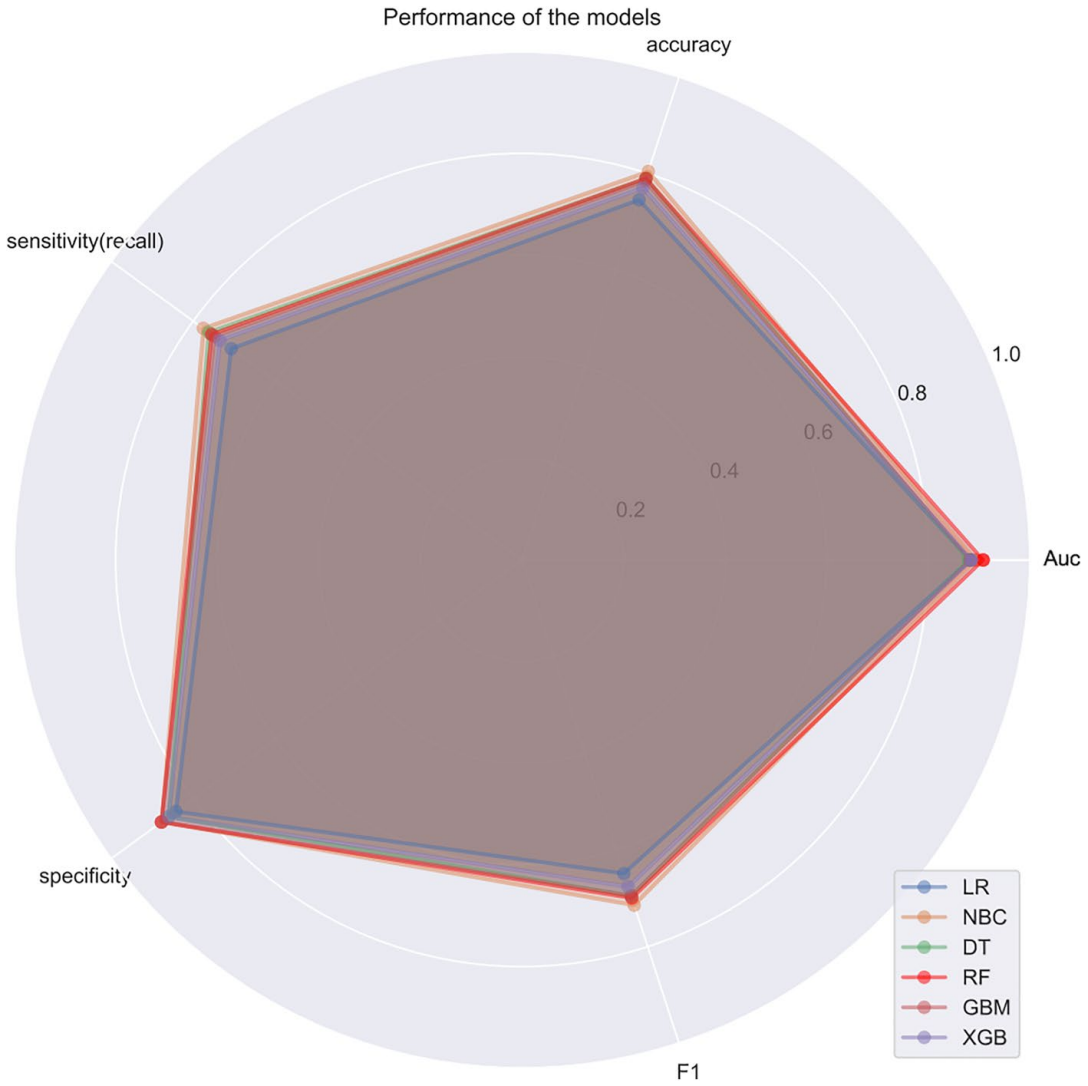
Performance of different models.

**Figure 4:**
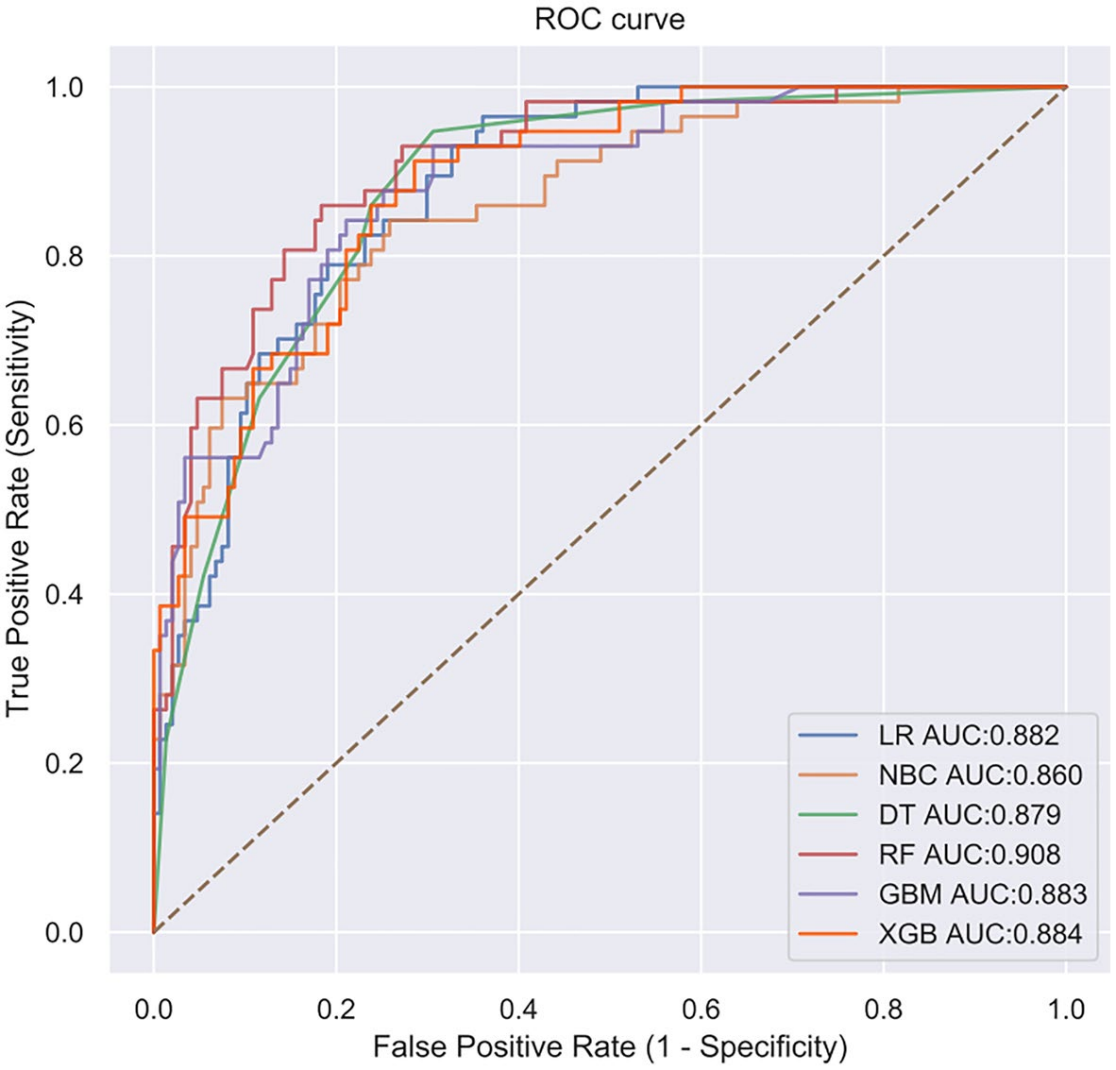
The results of the validation in the validation cohort.

Based on the six significant risk factors identified through multivariable Cox regression analysis on the training cohort, the following six machine learning algorithms were used to develop predictive models: Naive Bayesian classification (NBC), extreme Gradient Boosting (XGB), Random Forest (RF), Decision Tree (DT), Gradient Boosting Machine (GBM), and Logistic Regression (LR). For internal validation, tenfold cross-validation was employed to compare the performance of all models. ROC curves were plotted, and the AUCs of all established models are illustrated in Fig. 2. Among them, the RF model exhibited the best prediction performance with an AUC of 0.931. The AUCs of the XGBboost, GBM, DT, LR, and NBC models were 0.922, 0.923, 0.923, 0.829, and 0.871, respectively. Additionally, the selected models were comprehensively evaluated by calculating their accuracy, sensitivity, and specificity, as depicted in Fig. 3. Consequently, the RF model was hereby identified as the most effective predictive model for the prognosis of AIS, displaying high accuracy (0.789), sensitivity (0.755), and specificity (0.877). The F1 score is a commonly used metric for evaluating the overall performance of classification models, especially in situations with imbalanced class distributions. The F1 score ranges between zero and one, with values closer to one indicating better model performance. In the case of the final RF model, the F1 score was 0.699. Additionally, an external cohort was used to test the model, and the result also suggested that the RF model is the optimal model to predict the prognosis, with the highest ACU of 0.908. The AUCs for each model were: 0.884 for the XGB model, 0.883 for the GBM model, 0.879 for the DT model, 0.882 for the LR model, and 0.860 for the NBC model (Fig. 4).

### Model interpretation

**Figure 5:**
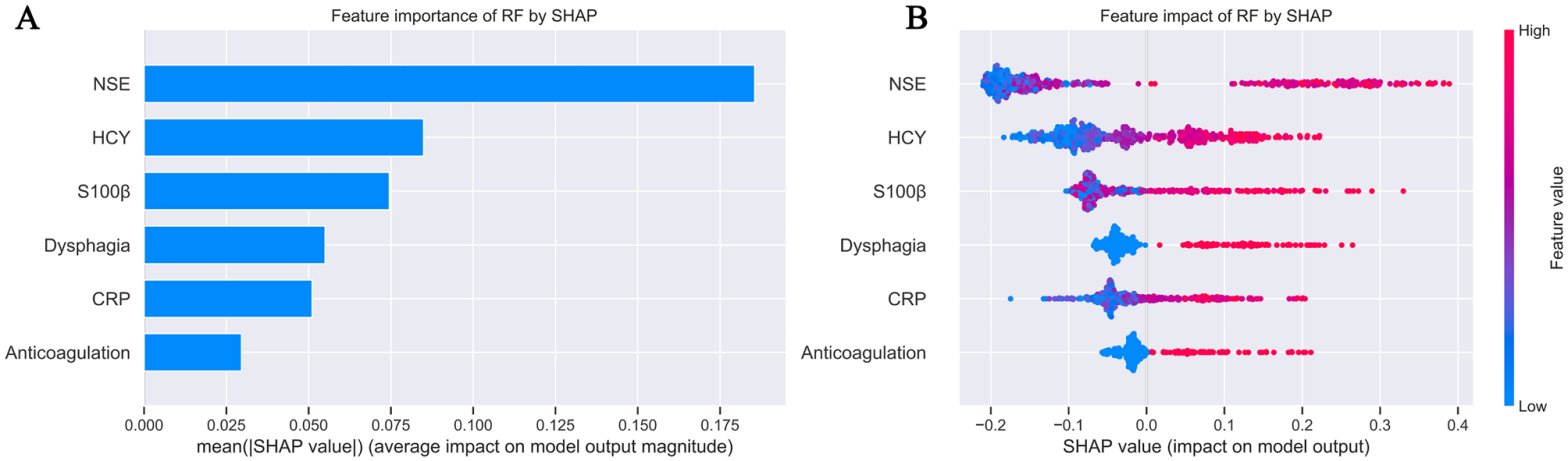
SHAP for the RF model. (**A**) importance ranking of features. (**B**) the distribution of the impacts of each feature on the model output.

This study analyzed the independent validation set in the RF model through the SHAP package, as shown in Fig. 5**.** In the final RF model, feature importance rankings of six predictors are shown in Fig. 5A**.** Based on the SHAP summary plots for poor prognosis in AIS patients, the related features ranked from highest to lowest importance were NSE, HCY, S-100β, dysphagia, CRP and anticoagulation. Figure 5B showed the distribution of the contribution of each feature to the model output. The influence of feature values on the results is represented by colors, with each dot representing a case in each row, red dots representing larger feature values, and blue representing lower ones. The smaller the feature value, the corresponding SHAP value is less than zero7, indicating a negative impact. On the other hand, the larger the feature value, the corresponding SHAP value is greater than zero, indicating a positive impact. The region with the widest distribution is NSE, indicating that it has the greatest impact.

### Online application for prognosis prediction

Based on the RF model, an easy-to-use online calculator was built to predict the prognosis of AIS patients, which can be obtained at https://mlmedicine-prog-prog-stroke-ysnamt.streamlitapp.com/. By entering the patient's clinical data, doctors and patients can obtain the estimated probability of poor prognosis immediately. Readers can also use these detailed parameter settings to reproduce the proposed model in Python. The *model_parameter_settings.txt* can be downloaded from the following publicly available GitHub repositories. (https://github.com/Wu-Shi-Nan/Acute-ischemic-stroke/blob/main/model_parameter_settings.txt).

## Discussion

Stroke is the second most common cause of disability and the leading cause of death among adults^13^, imposing a substantial burden on families and society in terms of disease and medical impact. Furthermore, this burden has consistently increased over the past three decades^14,15^. Consequently, there is an urgent need to research early and accurate prediction of adverse outcomes in patients with AIS, to facilitate precise clinical management and surveillance.

According to the present study, dysphagia was identified as a significant cause of poor prognosis in patients with stroke. Dysphagia is a common complication after a stroke^16^. Martino et al. reported that dysphagia occurs in 37% to 78% of patients with stroke and increases the risk for pneumonia 3–11 times in patients with confirmed aspiration^17^. Additionally, previous literature has proved that dysphagia is the most important cause of post-stroke pneumonia^18–20^. Stroke inhibits immunological responses through the activation of the autonomic nervous system and stress axis, contributing to the development of stroke-associated pneumonia, further exacerbating the condition^21^.

The present findings showed that CRP has a negative effect on the prognosis in stroke patients. CRP is an inflammatory response indicator, which can be significantly elevated during the acute phase of an inflammatory response. Meanwhile, stroke and infection are strongly intertwined. Many studies have reported that impaired immune response occurs in stroke patients, resulting in increased susceptibility to infections^22^. It has been previously reported that the presence of infection during acute and subacute phases of stroke is a common predictor of a poorer prognosis^23^. Higher CRP values as associated with worse the infection^24^. Increased c-reactive protein is related to a poorer prognosis regarding the course of stroke^23^.

Meanwhile, AIS patients are prone to develop inflammatory response, and the immune-inflammatory response is an essential process post-stroke^25^. Xie et al. showed that the interaction between the activation of coagulation and the inflammatory response leads to the consumption of coagulation factors. Additionally, patients with a higher international normalized ratio had more serious strokes, and the increased international normalized ratio was an independent predictor of one-year all-cause mortality^26^. Anticoagulation increases the international normalized ratio and bleeding risk, thus increasing the incidence of poor prognosis. Bautista et al. suggested that warfarin use at baseline was associated with mortality^27^, which is consistent with the findings of this study.

S100β and NSE were positively associated with poor prognosis in the present findings. S-100β, a member of a family of Ca + binding proteins, is a small acidic calcium-binding protein and abundantly expressed in the nervous system, mostly in astrocytes and several neuronal populations^28^. Clinically, it is considered to be a serum marker of cerebral damage and hypoxia for assessing neurological prognosis^29^. NSE, a dimeric isoenzyme of the glycolytic enzyme enolase, is involved in the glycolytic pathway. It is abundantly present in neurons and cells of neuroendocrine origin and used as a serum marker for neuronal loss, and its highest activity is found in brain tissue cells^30,31^. S100β and NSE are markers of glial cells^32^. Due to stroke, ischemia and hypoxia occur in the brain, leading to rupture of the membrane structure of the neurons, and the process of acute stroke causes a large release of S100β and NSE into the blood. The degree of neurological impairment can be assessed to some extent by S100β and NSE^23,33^. Previous studies have reported that increased levels of NSE and S100B are positively correlated with poor outcomes of stroke patients^34–36^, which is consistent with our findings. NSE and S100β were also included in the presented predictive model to evaluate the prognosis of patients with stroke. Meanwhile, NSE ranked first among all predictors based on SHAP analysis. In addition, HCY was related to a poor outcome in stroke patients, and the relationship between hyperhomocysteinemia and poor outcome in stroke patients has been previously reported^37^. When stroke patients presented with inflammatory response, cellular injury and necrosis would occur. Furthermore, as a response to this reaction, adenosine triphosphate would be released into the extracellular space, and hydrolyzed into adenosine. Adenosine has anti-inflammatory and tissue-protective properties^38^. However, high HCY could reduce activities and protein content of electron transport chain components, hence lowering mitochondrial energy metabolism and decreasing adenosine triphosphate production^39^. Thereby, for patients with high HCY, the anti-inflammatory and tissue protection in AIS patients were worse, and the prognosis was poorer accordingly.

**Figure 6:**
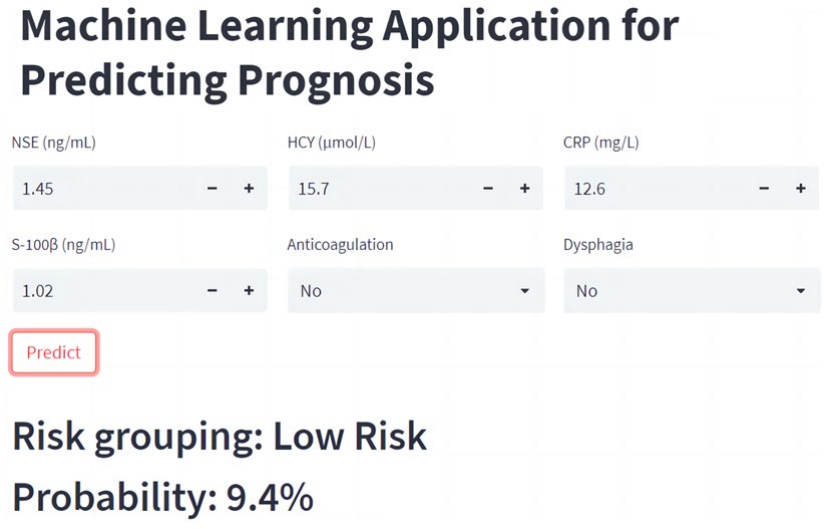
The web calculator for predicting prognosis of AIS patients.

An RF model was developed in this study to predict the risk of poor prognosis in AIS patients, which showed excellent performance compared with other ML models. The factors NSE, HCY, CRP, S-100β, anticoagulation, and dysphagia were identified as independent factors for poor prognosis. This study has laid a foundation for timely and accurate prediction of poor prognosis risk in order to further organize stroke care. Finally, poor prognosis rates for AIS patients can be easily obtained by entering corresponding clinical features in the developed online calculator. As shown in Fig. 6, the probability is calculated online quickly (Probability of poor prognosis = 9.4%, low-risk group). The wide application of smart devices nowadays makes the developed tool greatly convenient to use.

Previous studies have attempted to use ML algorithms to evaluate the prognosis of stroke^5,40–43^. Peng et al. leveraged a cohort of 423 patients for predicting 30-day mortality in spontaneous intracerebral hemorrhage patients^42^. Bacchi et al. designed models using LR, RF, DT, and artificial neural networks to predict in-hospital mortality^43^. Chen et al. used five ML techniques, including CatBoost, XGB, Boosting Decision Tree, RF, and AdaBoost, to investigate the 90-day poor prognosis in patients with transient ischemic attack or minor stroke. The factors associated with poor prognosis were identified, and the CatBoost model (AUC = 0.839) had the best predictive performance^40^. However, their study had a relatively short predictive window time. Heo et al. explored the applicability of ML algorithms to predict long-term prognosis in AIS patients. They reported that the deep neural network with an AUC of 0.888 can improve the prediction of long-term outcomes, but the predictive variables were not identified^5^.

Compared to other studies, the proposed predictive model has the following advantages. First, a comprehensive range of ML algorithms were tested, including NBC, XGB, RF, DT, GBM, and LR. This comprehensive approach allows exploration of different modeling techniques and selecting the most suitable algorithm for the specific prediction task. Second, several significant risk factors were identified through the analysis, such as NSE, HCY, CRP, S-100β, anticoagulation, and dysphagia, which are independently associated with unfavorable outcomes in AIS patients. The variables mentioned above are mostly readily available patient information, which makes this model potentially suitable for assisting clinical applications. Proper use of the model can provide valuable insights into the factors influencing prognosis and aids in clinical decision-making. Third, this study employed a relatively long prediction window, which extends the observation period and captures a broader range of patient outcomes. This reduces the impact of short-term fluctuations and random variability, enhancing the reliability and stability of the predictions. Fourth, the SHAP values were used to interpret the model, providing insights into the contribution and importance of each feature in the prediction process. This enhances the model transparency and interpretability, allowing for better understanding and trust in the results. Fifth, a web-based platform that visualizes the predictive model and provides a dynamic calculator for easy and practical usage was developed. This user-friendly interface enhances the accessibility and usability of the proposed model, making it more convenient for clinical applications. Overall, the predictive model stands out by utilizing clinical samples combined with various ML algorithms, identifying significant risk factors, employing a long prediction window, providing model interpretation, and offering a user-friendly platform. These advantages contribute to its potential clinical utility and effectiveness in predicting AIS outcomes.

There are several limitations present in this study. First, this is a retrospective study, which introduces the potential for selection bias and limits the establishment of causal relationships. Second, the sample size was not large enough, which may affect the generalization ability of the findings, and the data collection was limited to a single medical center, which may restrict the external validity of the results. Despite the good predictive power of our ML model, external validation in a completely different patient cohort is unavailable. Third, it is recommended to compare the predictive model with another prognostic system, such as the Acute Stroke Registry and Analysis of Lausanne score, to provide a more comprehensive evaluation and enhance the persuasiveness of the findings^44^. Finally, it should be noted that the NIHSS score, which stands for National Institutes of Health Stroke Scale, is a widely predictor of outcomes in AIS predictive models^45^. The NIHSS score upon admission was found to be a significant factor in the development of unfavorable outcomes. It is important to acknowledge that the absence of the NIHSS score data in the final predictive model might have limited its performance. A prospective multiple-center cohort could be designed to enhance the credibility of the results in the future.

## Conclusion

In summary, this paper established machine learning models to predict the prognosis of acute ischemic stroke. This tool can assist clinicians and patients to predict prognoses and formulate management strategies.

## Data Availability

Corresponding authors can be contacted for data upon reasonable request.

## References

[1] Mozaffarian D, Benjamin EJ, Go AS, Arnett DK, Blaha MJ, Cushman M, de Ferranti S, Despres JP, Fullerton HJ, Howard VJ (2015). Heart disease and stroke statistics–2015 update: A report from the American Heart Association. Circulation.

[2] Feigin VL, Nguyen G, Cercy K, Johnson CO, Alam T, Parmar PG, Abajobir AA, Abate KH, Abd-Allah F, Collaborators GBDLRoS (2018). Global, regional, and country-specific lifetime risks of stroke, 1990 and 2016. N. Engl. J. Med..

[3] Kim AS, Cahill E, Cheng NT (2015). Global stroke belt: Geographic variation in stroke burden worldwide. Stroke.

[4] Han H, Liu W (2019). The coming era of artificial intelligence in biological data science. BMC Bioinform..

[5] Heo J, Yoon JG, Park H, Kim YD, Nam HS, Heo JH (2019). Machine learning-based model for prediction of outcomes in acute stroke. Stroke.

[6] Lee KC, Hsu CC, Lin TC, Chiang HF, Horng GJ, Chen KT (2022). Prediction of prognosis in patients with trauma by using machine learning. Medicina (Kaunas).

[7] Li C, Liu M, Li J, Wang W, Feng C, Cai Y, Wu F, Zhao X, Du C, Zhang Y (2022). Machine learning predicts the prognosis of breast cancer patients with initial bone metastases. Front. Public Health.

[8] Chen S, Guo T, Zhang E, Wang T, Jiang G, Wu Y, Wang X, Na R, Zhang N (2022). Machine learning-based prognosis signature for survival prediction of patients with clear cell renal cell carcinoma. Heliyon.

[9] Stroke--1989: Recommendations on stroke prevention, diagnosis, and therapy. Report of the WHO Task Force on Stroke and other Cerebrovascular Disorders. *Stroke* 1989, 20:1407–1431. 10.1161/01.str.20.10.1407 2799873

[10] Güleç A, Albayrak I, Erdur Ö, Öztürk K, Levendoglu F (2021). Effect of swallowing rehabilitation using traditional therapy, kinesiology taping and neuromuscular electrical stimulation on dysphagia in post-stroke patients: A randomized clinical trial. Clin. Neurol. Neurosurg..

[11] Lundberg SM, Erion G, Chen H, DeGrave A, Prutkin JM, Nair B, Katz R, Himmelfarb J, Bansal N, Lee SI (2020). From local explanations to global understanding with explainable AI for trees. Nat. Mach. Intell..

[12] Nohara YMK, Soejima H, Nakashima N (2022). Explanation of machine learning models using shapley additive explanation and application for real data in hospital. Comput. Methods Progr. Biomed..

[13] Roth GA, Abate D, Abate KH, Abay SM, Abbafati C, Abbasi N, Abbastabar H, Abd-Allah F, Abdela J, Abdelalim A (2018). Global, regional, and national age-sex-specific mortality for 282 causes of death in 195 countries and territories, 1980–2017: A systematic analysis for the Global Burden of Disease Study 2017. Lancet.

[14] Collaborators GBDS (2021). Global, regional, and national burden of stroke and its risk factors, 1990–2019: A systematic analysis for the Global Burden of Disease Study 2019. Lancet Neurol..

[15] Winstein CJ, Stein J, Arena R, Bates B, Cherney LR, Cramer SC, Deruyter F, Eng JJ, Fisher B, Harvey RL (2016). Guidelines for adult stroke rehabilitation and recovery: A guideline for healthcare professionals from the American Heart Association/American Stroke Association. Stroke.

[16] Dziewas R, Stellato R, van der Tweel I, Walther E, Werner CJ, Braun T, Citerio G, Jandl M, Friedrichs M, Notzel K (2018). Pharyngeal electrical stimulation for early decannulation in tracheotomised patients with neurogenic dysphagia after stroke (PHAST-TRAC): A prospective, single-blinded, randomised trial. Lancet Neurol..

[17] Martino R, Foley N, Bhogal S, Diamant N, Speechley M, Teasell R (2005). Dysphagia after stroke: Incidence, diagnosis, and pulmonary complications. Stroke.

[18] Smith CJ, Kishore AK, Vail A, Chamorro A, Garau J, Hopkins SJ, Di Napoli M, Kalra L, Langhorne P, Montaner J (2015). Diagnosis of stroke-associated pneumonia: Recommendations from the pneumonia in stroke consensus group. Stroke.

[19] Armstrong JR, Mosher BD (2011). Aspiration pneumonia after stroke: Intervention and prevention. Neurohospitalist.

[20] Finlayson O, Kapral M, Hall R, Asllani E, Selchen D, Saposnik G, Canadian Stroke N (2011). Stroke Outcome Research Canada Working G: Risk factors, inpatient care, and outcomes of pneumonia after ischemic stroke. Neurology.

[21] Hotter B, Hoffmann S, Ulm L, Montaner J, Bustamante A, Meisel C, Meisel A (2020). Inflammatory and stress markers predicting pneumonia, outcome, and etiology in patients with stroke: Biomarkers for predicting pneumonia, functional outcome, and death after stroke. Neurol. Neuroimmunol. Neuroinflamm..

[22] Shi K, Wood K, Shi FD, Wang X, Liu Q (2018). Stroke-induced immunosuppression and poststroke infection. Stroke Vasc. Neurol..

[23] Lasek-Bal A, Jedrzejowska-Szypulka H, Student S, Warsz-Wianecka A, Zareba K, Puz P, Bal W, Pawletko K, Lewin-Kowalik J (2019). The importance of selected markers of inflammation and blood-brain barrier damage for short-term ischemic stroke prognosis. J. Physiol. Pharmacol..

[24] Pathak A, Agrawal A (2019). Evolution of C-reactive protein. Front. Immunol..

[25] Anrather J, Iadecola C (2016). Inflammation and stroke: An overview. Neurotherapeutics.

[26] Xie X, Wang X, Li Z, Zhao X, Miao Z, Liu L, Li H, Meng X, Wang Y, Wang Y (2019). Prognostic value of international normalized ratio in ischemic stroke patients without atrial fibrillation or anticoagulation therapy. J. Atheroscler. Thromb..

[27] Bautista AF, Lenhardt R, Yang D, Yu C, Heine MF, Mascha EJ, Heine C, Neyer TM, Remmel K, Akca O (2019). Early prediction of prognosis in elderly acute stroke patients. Crit. Care Explor..

[28] Donato R, Sorci G, Riuzzi F, Arcuri C, Bianchi R, Brozzi F, Tubaro C, Giambanco I (2009). S100B's double life: Intracellular regulator and extracellular signal. Biochim. Biophys. Acta.

[29] Stroick MFM, Ragoschke-Schumm A, Fassbender K, Bertsch T, Hennerici MG (2006). Protein S-100B—A prognostic marker for cerebral damage. Curr. Med. Chem..

[30] Shen QQ, Wang W, Wu H, Tong XW (2022). The effect of edaravone combined with DL-3-N-butylphthalide on the levels of tumor necrosis factor-alpha, interleukin-10, neuron-specific enolase and effect in patients with acute cerebral infarction. J. Physiol. Pharmacol..

[31] Jauch EC, Lindsell C, Broderick J, Fagan SC, Tilley BC, Levine SR (2006). Group Nr-PSS: Association of serial biochemical markers with acute ischemic stroke: The National Institute of Neurological Disorders and Stroke recombinant tissue plasminogen activator Stroke Study. Stroke.

[32] Rashwan HM, Mohammed HE, El-Nekeety AA, Hamza ZK, Abdel-Aziem SH, Hassan NS, Abdel-Wahhab MA (2021). Bioactive phytochemicals from Salvia officinalis attenuate cadmium-induced oxidative damage and genotoxicity in rats. Environ. Sci. Pollut. Res. Int..

[33] Kanavaki A, Spengos K, Moraki M, Delaporta P, Kariyannis C, Papassotiriou I, Kattamis A (2017). Serum levels of S100b and NSE proteins in patients with non-transfusion-dependent thalassemia as biomarkers of brain ischemia and cerebral vasculopathy. Int. J. Mol. Sci..

[34] Bloomfield SM, McKinney J, Smith L, Brisman J (2007). Reliability of S100B in predicting severity of central nervous system injury. Neurocrit. Care.

[35] Hu Y, Meng R, Zhang X, Guo L, Li S, Wu Y, Duan J, Ding Y, Ji X (2018). Serum neuron specific enolase may be a marker to predict the severity and outcome of cerebral venous thrombosis. J. Neurol..

[36] Kanazawa M, Takahashi T, Nishizawa M, Shimohata T (2017). Therapeutic strategies to attenuate hemorrhagic transformation after tissue plasminogen activator treatment for acute ischemic stroke. J. Atheroscler. Thromb..

[37] Forti P, Maioli F, Arnone G, Coveri M, Pirazzoli GL, Zoli M, Procaccianti G (2016). Homocysteinemia and early outcome of acute ischemic stroke in elderly patients. Brain Behav..

[38] Le TT, Berg NK, Harting MT, Li X, Eltzschig HK, Yuan X (2019). Purinergic signaling in pulmonary inflammation. Front. Immunol..

[39] Kaplan P, Tatarkova Z, Sivonova MK, Racay P, Lehotsky J (2020). Homocysteine and mitochondria in cardiovascular and cerebrovascular systems. Int. J. Mol. Sci..

[40] Chen SD, You J, Yang XM, Gu HQ, Huang XY, Liu H, Feng JF, Jiang Y, Wang YJ (2022). Machine learning is an effective method to predict the 90-day prognosis of patients with transient ischemic attack and minor stroke. BMC Med. Res. Methodol..

[41] Zhu Z, Xu T, Guo D, Huangfu X, Zhong C, Yang J, Wang A, Chen CS, Peng Y, Xu T (2018). Serum hepatocyte growth factor is probably associated with 3-month prognosis of acute ischemic stroke. Stroke.

[42] Peng SY, Chuang YC, Kang TW, Tseng KH (2010). Random forest can predict 30-day mortality of spontaneous intracerebral hemorrhage with remarkable discrimination. Eur. J. Neurol..

[43] Bacchi S, Oakden-Rayner L, Menon DK, Jannes J, Kleinig T, Koblar S (2020). Stroke prognostication for discharge planning with machine learning: A derivation study. J. Clin. Neurosci..

[44] Ntaios GFM, Ferrari J, Lang W, Vemmos K, Michel P (2012). An integer-based score to predict functional outcome in acute ischemic stroke: The ASTRAL score. Neurology.

[45] Chung CC, Su EC, Chen JH, Chen YT, Kuo CY (2023). XGBoost-based simple three-item model accurately predicts outcomes of acute ischemic stroke. Diagnostics (Basel).

